# Assessment of the Association Between Cigarette Smoking and Cognitive Performance in Patients With Schizophrenia-Spectrum Disorders: A Case-Control Study

**DOI:** 10.3389/fpsyt.2018.00642

**Published:** 2018-12-03

**Authors:** Filip Stramecki, Kamila D. Kotowicz, Patryk Piotrowski, Dorota Frydecka, Joanna Rymaszewska, Jan Aleksander Beszłej, Jerzy Samochowiec, Marcin Jabłoński, Michał Wroński, Ahmed A. Moustafa, Błazej Misiak

**Affiliations:** ^1^Department of Psychiatry, Wroclaw Medical University, Wroclaw, Poland; ^2^Department of Psychiatry, Pomeranian Medical University, Szczecin, Poland; ^3^School of Social Sciences and Psychology, Marcs Institute of Brain and Behaviour, Western Sydney University, Penrith, NSW, Australia; ^4^Department of Social Sciences, College of Arts and Sciences, Qatar University, Doha, Qatar; ^5^Department of Genetics, Wroclaw Medical University, Wroclaw, Poland

**Keywords:** schizophrenia, tobacco, smoking, nicotine, cognition

## Abstract

The prevalence of cigarette smoking is significantly higher in patients with schizophrenia compared to the general population. Schizophrenia is also characterized by cognitive impairments that can be detected in the premorbid phase of illness. However, studies addressing the association between cigarette smoking and cognition in patients with psychosis have provided mixed findings. Therefore, the aim of this study was to assess the relationship between tobacco smoking and cognitive performance in patients with schizophrenia. In this case-control study, we recruited 67 inpatients with schizophrenia (34 cigarette smokers) and 62 healthy controls (30 cigarette smokers) at two clinical sites (Wroclaw and Szczecin, Poland). Cognitive performance was examined using the Repeatable Battery for the Assessment of Neuropsychological Status (RBANS). Smoking dependence was determined using the Fagerström Test for Nicotine Dependence (FTND) and the pack-year index. Results show that, after adjustment for potential confounders, smokers with schizophrenia presented significantly lower scores on delayed memory tests compared to non-smokers with schizophrenia (*F* = 11.07, *p* = 0.002). In healthy controls, after adjustment for age, sex, and education level, smokers had significantly lower scores in immediate memory (47.1 ± 6.4 vs. 52.0 ± 4.0, *F* = 11.64, *p* = 0.001), visuospatial/constructional functions (34.8 ± 3.8 vs. 37.7 ± 1.8, *F* = 12.86, *p* = 0.001) and global cognition (177.0 ± 15.7 vs. 191.2 ± 14.0, *F* = 12.63, *p* = 0.001) compared to non-smokers. There were no significant correlations between FTND scores or pack-year index and cognitive performance neither in patient nor control group. Our results show that cigarette smoking is related to worse delayed memory performance in schizophrenia patients as well as deficits of immediate memory, visuospatial/constructional functions, and global cognition in controls. Longitudinal studies are required to establish causal interference between smoking and cognition in patients with schizophrenia.

## Introduction

Schizophrenia is a severe mental illness resulting from complex interactions between genetic and environmental factors (1). Several studies indicate a high prevalence of tobacco smoking in patients with schizophrenia that has been estimated at around 58–88%, which is significantly higher than in the general population (23%) (2). There are many hypotheses attempting to explain this observation. For instance, it has been proposed that cigarette smoking is a “self-medication” process, by which the patients cope with the negative symptoms of the disease, compensating for neurotransmission abnormalities in the central nervous system (3). Based on this hypothesis, patients might also smoke to alleviate the extrapyramidal side effects associated with antipsychotic treatment since nicotine induces the 1A2 isoform of cytochrome P450 (CYP1A2) that is involved in metabolizing a number of antipsychotic drugs (4, 5). Finally, it has been proposed that the occurrence of schizophrenia and nicotine dependence are related to overlapping genetic backgrounds and environmental factors (6).

Several studies show that smoking among patients with schizophrenia has a significant impact on the psychopathological manifestation of the disease. However, these studies have provided mixed results showing either higher, lower or similar severity of positive and negative symptoms in smokers compared to non-smokers (7–12). Similarly, there is some evidence that smoking might impact cognitive performance in schizophrenia patients. It is widely known that cognitive deficits represent one of the main elements of schizophrenia psychopathology and are present in the majority of patients, largely influencing functional outcomes (13, 14). Cognitive impairments in schizophrenia include, among others, deficits in verbal learning, memory, attention, working, and visuospatial memory as well as processing speed (15). There is a growing body of evidence suggesting that cigarette smoking might also impact cognitive performance in patients with schizophrenia. However, studies addressing this link between cigarette smoking and cognition have provided mixed results. Some studies have shown that cigarette-smoking patients might present with better cognitive performance compared to non-smokers with schizophrenia (16). It has also been reported that smoking cessation in patients with schizophrenia might result in the deterioration of visuospatial memory and attentional deficits with subsequent improvement in cognition after re-starting tobacco smoking (17).

However, it has also been shown that tobacco smoking might be associated with worse cognitive performance in schizophrenia patients, especially in the domain of visuospatial memory (18, 19). Finally, several studies revealed that there is no significant association between tobacco smoking and cognition in schizophrenia (10, 20, 21). Interestingly, studies investigating the impact of tobacco smoking in non-clinical populations have provided more consistent results. It has been shown that young adults, who are addicted to nicotine experienced significantly higher deficits in attention and greater visuospatial working memory impairments compared to non-smokers (22, 23).

Interestingly, experimental studies have revealed that intranasal nicotine administration might improve spatial organization and memory in patients with schizophrenia (17, 24, 25). Moreover, transdermal nicotine administration has been found to improve memory performance (24, 26). However, the effects of short-term nicotine administration do not equal long-term cohort studies in cigarette smokers. The opposite findings of these studies can results from the multifactorial etiology of cognitive disturbances. The impairments may be caused not exclusively by nicotine, but by a large number of cytotoxic compounds present in a cigarette smoke and cause adverse effects in the brain (27). In general, large long-term cohort studies by Depp et al. (19) and Vermeulen et al. (27) have reported poorer cognitive functioning in cigarette smokers with psychotic disorders.

As mentioned above, studies addressing the impact of cigarette smoking on cognition have provided mixed findings. The majority of these studies have been performed in multiple-episode schizophrenia patients and have not included a group of healthy controls. Therefore, the aim of this study was to investigate the association between cigarette smoking and cognitive functioning in patients with schizophrenia and in the group of healthy controls.

## Materials and methods

### Participants

Participants were 67 patients with schizophrenia-spectrum disorders (34 smokers and 33 non-smokers) and 62 healthy controls (30 smokers and 32 non-smokers). There were following inclusion criteria for the patients: (1) age between 18 and 65 years and (2) a diagnosis of schizophrenia-spectrum disorders according to the DSM-IV criteria. The exclusion criteria for the patients were as follows: (1) co-occurrence of neurological disorders; (2) intellectual disability; (3) drug and alcohol dependence (except for nicotine dependence). In the healthy control group, the exclusion criteria included also psychiatric treatment and the presence of past, present, or family history of neurological and psychiatric disorders (except for nicotine dependence). There was no history of cardiovascular diseases, diabetes, and hypertension in the group of patients and controls.

All patients were recruited at Lower Silesian Center of Mental Health (Wroclaw, Poland), Department of Psychiatry (Wroclaw Medical University, Wroclaw, Poland) and Department of Psychiatry (Pomeranian Medical University, Szczecin, Poland) in the years 2016–2018. A diagnosis of schizophrenia-spectrum disorders was established using the DSM-IV criteria and validated with the Operational Criteria for Psychotic Illness (OPCRIT) checklist (28). There were 17 multiple-episode schizophrenia patients and 50 first-episode psychosis (FEP) patients. The latter group represented the following diagnoses: schizophrenia (*n* = 28), schizophreniform disorder (*n* = 14), brief psychotic disorder (*n* = 4), schizoaffective disorder (*n* = 3) and delusional disorder (*n* = 1). Current psychopathological manifestation was assessed using the Positive and Negative Syndrome Scale (PANSS), which consists of three subscales: positive symptoms, negative symptoms, and general psychopathology (29). Based on a differential receptor affinity profiles of antipsychotics (30, 31), patients were divided into three groups: (1) those receiving antipsychotics with high anticholinergic activity: clozapine, olanzapine, chlorpromazine, zuclopenthixol, levopromazine, and perazine (*n* = 30); (2) those receiving antipsychotics with low anticholinergic activity: amisulpride, aripiprazole, haloperidol, risperidone, sertindol, sulpride, quetiapine, and ziprasidone (*n* = 23) and (3) those receiving at least two antipsychotics with opposite anticholinergic activity (*n* = 14). Healthy controls were recruited at the Wroclaw Medical University through advertisements. Patients and controls were selected as a convenience sample of individuals with data regarding cigarette smoking and cognitive performance. Both groups were matched for age, sex, and cigarette smoking status.

Tobacco smoking dependence was assessed with the use of the Fagerström Test for Nicotine Dependence (FTND) (32) and the pack-year index (33). Patients were classified as smokers if they reported smoking more than one cigarette per day for at least 12 months. Cognitive performance of all participants in the study was examined using the Repeatable Battery for the Assessment of Neuropsychological Status (RBANS) (34). The RBANS has been validated in several studies of patients with schizophrenia, showing good psychometric properties (35–37). The RBANS captures the following cognitive domains: (1) immediate memory (measured by word List Learning and Story Memory test), (2) visuospatial and constructional working memory (Figure Copy and Line Orientation), (3) language capacity (Picture naming and Semantic Fluency), (4) attention (Digit Span and Coding), and (5) delayed memory (List Recall, List Recognition, Story Memory, and Figure Recall). Assessment of cognition was performed by psychologists, who were blind to the cigarette smoking status of participants. The FTND and questions regarding cigarette smoking status were administered after assessment of cognitive performance.

The study protocol was approved by the Ethics Committee at Wroclaw Medical University (Wroclaw, Poland) and followed the Declaration of Helsinki of ethical principles for human research. All participants gave a written informed consent.

### Statistical analysis

The normality of data distribution was tested using the Kolmogorov–Smirnov test. Due to non-normal distribution of continuous variables, non-parametric tests were used to perform bivariate comparisons between the groups. Similarly, correlations were assessed using the Spearman's rank correlation coefficients. Distribution of categorical variables in patients and controls was compared using the χ^2^ test. The analysis of co-variance (ANCOVA) was performed to test the effects of cigarette smoking status on cognitive performance after co-varying for age, sex, education level, and the chlorpromazine equivalent dosage (CPZeq). In the case of patients, the stage of illness (FEP patients or multiple-episode patients) was also added as a co-variate. We included scores of cognitive performance on domains that differed significantly between patients and controls as dependent variables in the ANCOVA. Before running the ANCOVA, scores of cognitive performance were transformed to Z-scores because of non-normal distribution. The results of statistical analysis were considered significant if *p*-value was < 0.05. Statistical analysis was performed using the Statistical Package for Social Sciences, version 20 (SPSS Inc., Chicago, Illinois, USA).

## Results

Scores of all cognitive domains and education level were significantly lower in the group of patients compared to controls (Table 1). General characteristics of patients and controls with respect to cigarette smoking status are shown in Table 2. All subgroups of participants did not differ significantly in terms of age and sex. The level of education was significantly lower in smokers compared to non-smokers. There were no significant differences in the FTND score and pack-year index between smoking patients and smoking controls.

**Table 1:** General characteristics of patients and controls.

	**Patients (*n* = 67)**	**Controls (*n* = 62)**	***p***
Age, years	30.3 ± 9.7	30.5 ± 6.4	0.349
Sex, M/F (%)	36 (53.7)/31 (46.3)	28 (45.2)/34 (54.8)	0.331
Education, higher/other than higher (%)	18 (26.9)/49 (73.1)	5 (8.1)/57 (91.9)	**0.005**
FTND score	2.4 ± 2.9	2.1 ± 3.1	0.535
Pack-year index	5.6 ± 8.8	4.1 ± 6.9	0.554
Immediate memory	40.5 ± 10.9	49.7 ± 5.8	<**0.001**
Visuospatial/ constructional functions	33.6 ± 5.4	36.3 ± 3.3	**0.005**
Language	27.8 ± 6.1	31.8 ± 5.8	<**0.001**
Attention	49.4 ± 13.7	63.2 ± 10.8	<**0.001**
Delayed memory	42.9 ± 9.8	53.1 ± 4.6	<**0.001**
Global cognition	153.6 ± 28.6	184.3 ± 16.4	<**0.001**

*FTND, the Fagerström Test for Nicotine Dependence. Data expressed as mean ± SD or the number of cases. Raw scores of cognitive performance are provided. Significant differences were marked with bold characters (p < 0.05)*.

**Table 2:** General characteristics of schizophrenia patients and healthy controls with respect to cigarette smoking status.

	**SZ-S (*n* = 34)**	**SZ-NS (*n* = 33)**	**HCs-S (*n* = 30)**	**HCs-NS (*n* = 32)**	***p***
Age, years	31.6 ± 9.6	29.0 ± 9.8	30.9 ± 5.9	30.0 ± 6.8	0.251
Sex, M/F (%)	21 (61.8)/13 (38.2)	15 (45.4)/18 (54.6)	13 (43.3)/17 (56.7)	15 (46.9)/17 (53.1)	0.423
Education, higher/other than higher (%)	5 (14.7)/29 (85.3)	13 (39.4)/20 (60.6)	1 (3.3)/29 (96.7)	4 (12.5)/28 (87.5)	**0.001**
CPZeq, mg/day	362.8 ± 210.4	324.7 ± 239.9	–	–	0.284
PANSS-P	13.2 ± 4.9	14.7 ± 5.7	–	–	0.348
PANSS-N	20.2 ± 9.8	19.0 ± 8.0	–	–	0.773
PANSS-G	30.1 ± 9.5	31.8 ± 8.9	–	–	0.301
PANSS-total	61.6 ± 20.7	65.6 ± 19.1	–	–	0.486
FTND score	4.6 ± 2.4	–	4.5 ± 3.1	–	0.770
Pack-year index	11.1 ± 9.6	–	8.3 ± 8.1	–	0.238

*CPZeq, chlorpromazine equivalent dosage; FTND, the Fagerström Test for Nicotine Dependence; 12HCs-S, smoking controls; HCs-NS, non-smoking controls; PANSS-G, the Positive and Negative Syndrome Scale (a severity of general psychopathology); PANSS-N, the Positive and Negative Syndrome Scale (a severity of negative symptoms); PANSS-P, the Positive and Negative Syndrome Scale (a severity of positive symptoms); PANSS-total, the Positive and Negative Syndrome Scale (total score); SZ-NS, non-smokers with schizophrenia; SZ-S, smokers with schizophrenia*.

*Significant differences were marked with bold characters (p < 0.05)*.

Smokers with schizophrenia scored significantly lower on delayed memory compared to non-smokers with schizophrenia (Table 3). Smoking controls had significantly lower scores of immediate memory, visuospatial/constructional abilities, language, and global cognition compared to non-smoking controls.

**Table 3:** Cognitive performance with respect to cigarette smoking in patients with schizophrenia and healthy controls.

	**SZ-S**	**SZ-NS**	***p***	**HCs-S**	**HCs-NS**	***p***
Immediate memory	37.7 ± 8.6	43.3 ± 12.4	0.077	47.1 ± 6.4	52.0 ± 4.0	**0.001**
Visuospatial/constructional functions	33.0 ± 5.1	34.1 ± 5.8	0.232	34.8 ± 3.8	37.7 ± 1.8	**0.001**
Language	26.7 ± 6.0	28.8 ± 6.0	0.245	30.3 ± 5.4	33.2 ± 5.8	**0.049**
Attention	49.0 ± 12.5	49.8 ± 15.0	0.702	60.2 ± 9.9	66.0 ± 11.0	0.053
Delayed memory	40.2 ± 9.0	45.7 ± 9.8	**0.013**	51.7 ± 5.1	54.4 ± 3.7	0.059
Global cognition	148.9 ± 26.5	158.4 ± 30.3	0.146	177.0 ± 15.7	191.2 ± 14.0	**0.002**

*HCs-S, smoking controls; HCs-NS, non-smoking controls; SZ-NS, non-smokers with schizophrenia; SZ-S, smokers with schizophrenia. Raw scores are presented as mean ± SD. Significant differences were marked with bold characters (p < 0.05)*.

The results of ANCOVA testing for differences in cognitive performance between smokers and non-smokers, controlling for the effects of age, sex, education, stage of illness (FEP vs. multiple-episode patients), type of antipsychotics based on anticholinergic activity, and CPZeq are presented in Table 4. The ANCOVA test confirmed a significant association between cigarette smoking status and delayed memory performance in the group of patients. This ANCOVA test also revealed a significant association between sex and delayed memory. The association between cigarette smoking and immediate memory, visuospatial/constructional functions, as well as global cognition appeared to be significant in healthy controls after adjustment for age, sex, and education level. The ANCOVA model testing for differences in global cognition between smoking and non-smoking controls also demonstrated a significant association with age.

**Table 4:** ANCOVA results testing for differences in cognitive performance between smokers and non-smokers after co-varying for education level, global psychopathology, and medication effects.

	**Smoking (yes/no)**	**Age**	**Sex**	**Education level**	**CPZeq**	**Anticholinergic activity of antipsychotics (high vs. low vs. mixed)**	**Group (FEP vs. MES)**
Delayed memory, SZ patients	***F*** = **11.07** ***p*** = **0.002**	*F* = 1.26 *p* = 0.266	***F*** = **4.36** ***p*** = **0.041**	*F* = 0.60 *p* = 0.442	*F* = 0.24 *p* = 0.627	*F* = 0.53 *p* = 0.470	*F* = 3.28 *p* = 0.075
Immediate memory, HCs	***F*** = **11.64** ***p*** = **0.001**	*F* = 0.83 *p* = 0.366	*F* = 0.46 *p* = 0.500	*F* = 0.001 *p* = 0.978	–	–	–
Visuospatial/ constructional functions, HCs	***F*** = **12.86** ***p*** = **0.001**	*F* < 0.001 *p* = 0.999	*F* = 1.99 *p* = 0.163	*F* = 0.79 *p* = 0.377	–	–	–
Language, HCs	*F* = 3.30 *p* = 0.074	*F* = 0.27 *p* = 0.603	*F* = 1.51 *p* = 0.223	*F* = 0.503 *p* = 0.481	–	–	–
Global cognition, HCs	***F*** = **12.63** ***p*** = **0.001**	***F*** = **7.91** ***p*** = **0.007**	*F* = 0.17 *p* = 0.683	*F* = 0.36 *p* = 0.552	–	–	–

*CPZeq, chlorpromazine equivalent dosage; FEP, first-episode psychosis; MES, multiple-episode schizophrenia; PANSS, the Positive and Negative Syndrome Scale. Significant effects were marked with bold characters (p < 0.05)*.

There were no significant correlations between the FTND score or the pack-year index and cognitive performance neither in the group of smoking patients nor in smoking controls (Table 5).

**Table 5:** Correlations between FTND score, pack-year index cognitive performance in smokers.

	**SZ-S patients**		**HCs-S**	
	**FTND score**	**Pack-year index**	**FTND score**	**Pack-year index**
Immediate memory	*r* = 0.073 *p* = 0.681	*r* = −0.044 *p* = 0.807	*r* = 0.297 *p* = 0.125	*r* = −0.039 *p* = 0.843
Visuospatial/constructional functions	*r* = 0.026 *p* = 0.882	*r* = 0.008 *p* = 0.967	*r* = −0.187 *p* = 0.341	*r* = −0.099 *p* = 0.617
Language	*r* = 0.180 *p* = 0.309	r = −0.011 *p* = 0.949	*r* = 0.247 *p* = 0.205	*r* = −0.095 *p* = 0.630
Attention	*r* = 0.006 *p* = 0.973	*r* = 0.039 *p* = 0.830	*r* = −0.163 *p* = 0.408	*r* = −0.148 *p* = 0.454
Delayed memory	*r* = −0.023 *p* = 0.898	*r* = 0.044 *p* = 0.807	*r* = 0.186 *p* = 0.343	*r* = −0.092 *p* = 0.640
Global cognition	*r* = 0.102 *p* = 0.568	*r* = 0.022 *p* = 0.903	*r* = 0.009 *p* = 0.963	*r* = −0.163 *p* = 0.406

*FTND, the Fagerström Test for Nicotine Dependence; HCs-S, smoking controls; SZ-S, smokers with schizophrenia*.

## Discussion

This study investigated whether cognitive deficits observed in schizophrenia are associated with smoking behavior. We found worse performance of delayed memory in the group of smoking patients after adjustment for age, sex, educational attainment, illness stage, and medication effects. Further, smoking controls performed worse on immediate memory, visuospatial/constructional functions, and global cognition when compared to non-smoking healthy individuals. A severity of nicotine dependence was not related to the extent of cognitive impairments in our sample. The previous study by Zhang et al. (38), which was also based on the use of RBANS, revealed worse performance of visuospatial/constructional abilities, immediate memory and global cognition in male smokers with schizophrenia compared to non-smokers. Cigarette smoking has also been associated with impairments in attention (15), semantic fluency (39), visual learning (18), and global cognition (19). A large, prospective 6-year follow-up study by 26], revealed that cigarette smoking is related to worse processing speed in patients with non-affective psychosis. Other studies have revealed improved cognitive performance in smokers with schizophrenia (40) or a lack of association between nicotine dependence and cognition (20, 21).

Discrepancies across studies addressing the effects of cigarette smoking on cognition in schizophrenia patients can be attributed to several methodological differences. Firstly, it should be noted that patients were recruited during various stages of illness in the above-mentioned studies. Indeed, some studies were performed in FEP patients (20, 21, 41) while other studies assessed cognition in multiple-episode schizophrenia patients (15, 18, 39). Although the dosage of medications was controlled in the majority of these studies, antipsychotics largely differ in terms of pro-cognitive activity and side effects (42, 43). This is particularly related to the self-medication hypothesis. According to this theory, patients may unintentionally engage in smoking habits to increase the metabolism of antipsychotics and alleviate extrapyramidal symptoms or improve cognitive deficits and negative symptoms (44). Moreover, there is some evidence that treatment-resistance might be related to cognitive impairments in this group of patients (30). Another point is that various definitions of cigarette smoking have been used and a detailed history of smoking habits has not been recorded in most studies. In addition, a severity of nicotine dependence has not been controlled in a number of previous studies. Finally, schizophrenia is also associated with high prevalence rates of comorbid cardiovascular diseases that might further contribute to cognitive deficits (45, 46). A recent meta-analysis revealed that a diagnosis of metabolic syndrome together with its single components (central obesity, dyslipidaemia, diabetes, and hypertension) is related to cognitive impairment in patients with schizophrenia (47). Therefore, it might be assumed various prevalence rates of metabolic syndrome and related conditions in distinct studies might impact the association between cigarette smoking and cognition in schizophrenia.

The results of this study should be interpreted with caution, taking into account certain limitations. First, it should be noted that power analysis was not performed and our study had a relatively small sample size. Although this may suggest that our results could have occurred by coincidence, our findings are consistent with several previous studies. Second, our cross-sectional study design does not enable us to explain the reciprocal interactions between tobacco smoking and cognitive performance. Cigarette smoking might contribute to cognitive impairment via various mechanisms, including the effects on neurotransmission systems and vascular endothelium (48). However, certain cognitive deficits might make the patients more prone to engage in smoking behaviors. For instance, it has been reported that impairments in sustained attention and control of impulsivity may be a risk factor for cigarette smoking (49). Another limitation of our study is that the majority of our patients were not drug-naïve. However, the measures of antipsychotic treatment did not differ significantly between smoking and non-smoking patients and were included in the ANCOVA tests. In addition, our sample was not representative and thus it is difficult to generalize our findings over the entire population of patients. At this point, it is important to note that patients with schizophrenia and comorbid addictions other than nicotine dependence were excluded. Moreover, we did not record the use of other drugs that are frequently used by patients with psychotic disorders, such as cannabis. It also has to be noted that we did not take into account the influence of specific drugs on cognitive functions, different doses of these drugs, and the complex effect of drug-nicotine interaction on the symptoms of the disease. Finally, we did not control for a severity of extrapyramidal side effects that might be related to both cognitive deficits and cigarette smoking.

In summary, our study demonstrated that cigarette smoking is associated with impairments of delayed memory in patients with schizophrenia. However, in healthy, controls, cigarette smoking was related to worse performance of immediate memory, visuospatial/constructional abilities, and global cognition. The mechanisms underlying these differential associations in patients and controls remain unknown and require further studies. The association between cigarette smoking and cognitive impairment may not be related to the severity of nicotine dependence. Longitudinal studies are required to establish the direction of causality between cigarette smoking and cognition in schizophrenia.

## Author contributions

FS, DF, and BM contributed conception and design of the study. FS, KK, PP, DF, JB, JS, MJ, and MW performed clinical assessment of patients. FS organized the database. BM performed the statistical analysis. FS wrote the first draft of the manuscript. FS, BM, JR, and AM wrote sections of the manuscript. All authors contributed to manuscript revision, read, and approved the submitted version.

### Conflict of interest statement

The authors declare that the research was conducted in the absence of any commercial or financial relationships that could be construed as a potential conflict of interest.
